# In Situ Characterization of Anode Materials for Rechargeable Li-, Na- and K-Ion Batteries: A Review

**DOI:** 10.3390/ma19020280

**Published:** 2026-01-09

**Authors:** Jinqi Gui, Shuaiju Meng, Xijun Liu, Zhifeng Wang

**Affiliations:** 1 State Key Laboratory of Advanced Processing and Recycling of Non-Ferrous Metals, School of Materials Science and Engineering, Lanzhou University of Technology, Lanzhou 730050, China; 18830719390@163.com; 2 School of Resources, Environment and Materials, Guangxi University, Nanning 530004, China; 3 “The Belt and Road Initiative” Advanced Materials International Joint Research Center of Hebei Province, School of Materials Science and Engineering, Hebei University of Technology, Tianjin 300401, China

**Keywords:** in situ characterization, battery, anode, energy storage

## Abstract

Rechargeable lithium-, sodium-, and potassium-ion batteries are utilized as essential energy storage devices for portable electronics, electric vehicles, and large-scale energy storage systems. In these systems, anode materials play a vital role in determining energy density, cycling stability, and safety of various batteries. However, the complex electrochemical reactions and dynamic changes that occur in anode materials during charge–discharge cycles generate major challenges for performance optimization and understanding failure mechanisms. In situ characterization techniques, capable of real-time tracking of microstructures, composition, and interface dynamics under operating conditions, provide critical insights that bridge macroscopic performance and microscopic mechanisms of anodes. This review systematically summarizes the applications of such techniques in studying anodes for lithium-, sodium-, and potassium-ion batteries, with a focus on their contributions across different anode types. It also indicates current challenges and future directions of these techniques, aiming to offer valuable references for relevant applications and the design of high-performance anodes.

## 1. Introduction

The past three decades have witnessed rapid advances in electrochemical energy storage. While lithium-ion batteries have achieved remarkable commercial and academic success [1,2,3], this has spurred interest in alternative technologies. Sodium-ion batteries have re-emerged as a cost-effective solution with mitigated supply chain risks [4,5,6,7], and potassium-ion batteries have gained significant interest owing to their high natural abundance, competitive energy density, fast kinetics, and low cost [8,9]. A key tool driving the understanding of all three systems is in situ characterization, which is widely used to probe anode materials and monitor real-time battery behavior during operation [10].

In situ characterization techniques are critically important in the studies of anode materials. They overcome the limitations of traditional ex situ methods by dynamically and directly establishing the “structure–composition–performance” relationship of anode materials under real operating conditions. These techniques provide direct evidence for understanding intrinsic mechanisms behind core issues such as capacity fading and poor cycling stability [11]. Furthermore, they guide the design optimization and process improvement of anode materials toward higher capacity, longer lifespan, and enhanced safety, thereby accelerating the development and industrialization of novel anodes for energy storage applications [12,13,14].

**Figure 1:**
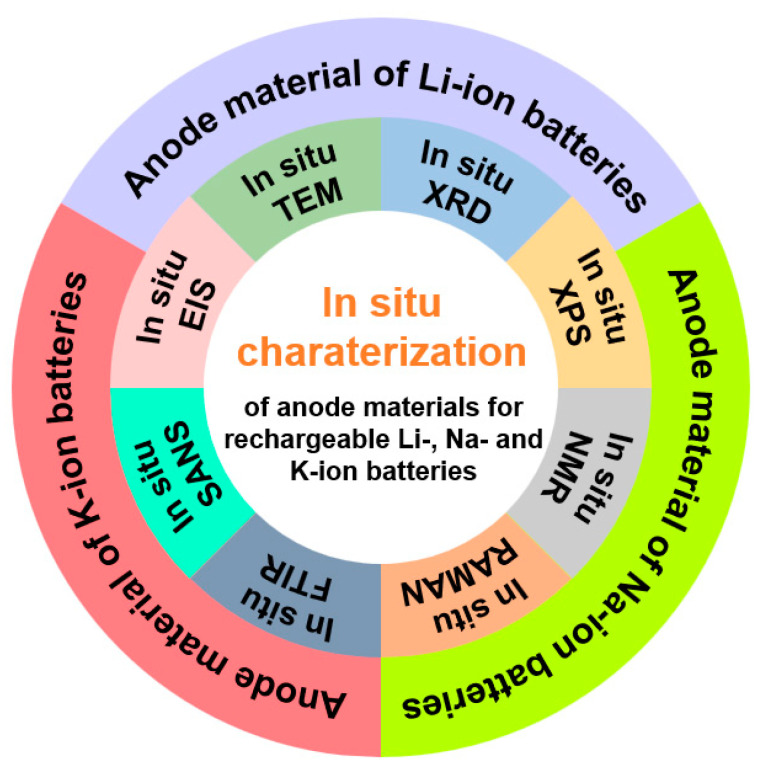
Scheme of in situ techniques for the studies of anode materials in Li-, Na- and K-ion batteries.

In recent years, in situ characterization techniques have been increasingly employed in the study of various anode materials (Figure 1). While several reviews have summarized the application of individual in situ techniques across different anodes, they often lack comprehensive coverage of multiple common methodologies [15]. Others have surveyed multiple characterization methods but focused only on a specific type or category of anode materials [16,17,18,19,20]. Although a limited number of reviews have discussed the use of multiple in situ techniques in anode studies, they are confined to a single battery type [21]. Therefore, a comprehensive review summarizing the applications of various in situ characterization techniques across different battery anode materials is urgently needed. Such work would help consolidate recent research findings and provide valuable guidance for future studies.

The current review systematically summarizes the applications of diverse in situ characterization techniques in the research of anode materials for lithium-ion, sodium-ion, and potassium-ion batteries, addressing the gap of insufficient comprehensive coverage in existing reviews. It first outlines the core advantages, spatial–temporal resolution, applicable configurations, contributions and limitations of eight mainstream in situ techniques. Subsequently, via specific research cases, it elaborates on how each technique reveals the electrochemical reaction mechanisms, dynamic structural evolution, interfacial behavior, and ion transport kinetics of different anode materials during charge–discharge cycles, and discusses the complementary application of combined techniques to overcome single-method limitations. Finally, the review summarizes current progress, identifies key challenges, and proposes forward-looking perspectives on future research directions. By integrating novel insights for anode performance optimization and structural design, this work aims to provide a practical reference for alkali metal ion battery researchers, accelerating the development of high-performance anode materials and related technologies.

## 2. In Situ Characterization Technique

In situ characterization techniques offer significant advantages in anode materials studies. Their core strength lies in the ability to track real-time changes in morphology, chemical composition, and interfacial states of anode materials under dynamic operating conditions that simulate or are found in real battery charge–discharge processes [22,23]. This capability overcomes the limitations of traditional ex situ methods, which often miss transient reaction stages and are susceptible to artifacts introduced during sample transfer. Table 1 systematically sorts out the characterization contributions, restrictions and applicable battery configurations of eight mainstream in situ techniques, clarifying their basic application characteristics [24]. It is worth noting that different characterization techniques have significant differences in terms of spatial and temporal resolution, the information obtained, and the physical parameters. Researchers need to select appropriate in situ characterization methods based on different detection purposes.

**Figure 2:**
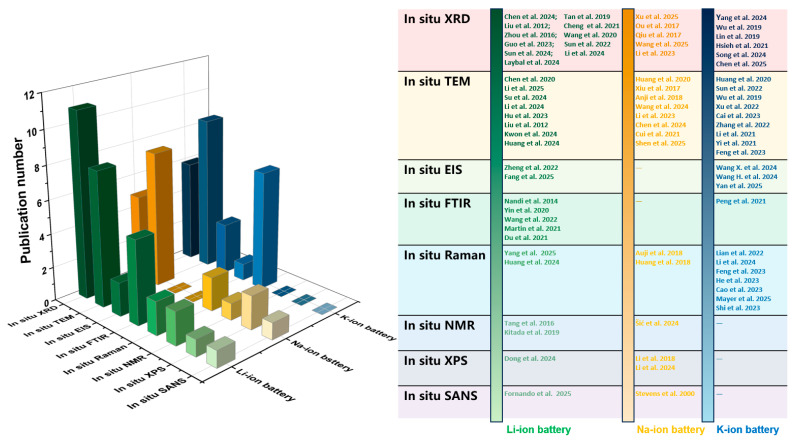
Statistics regarding application status of various in situ characterization techniques in anode material studies for Li-, Na-, and K-ion batteries in recent years [49,50,51,52,53,54,55,56,57,58,59,60,61,62,63,64,65,66,67,68,69,70,71,72,73,74,75,76,77,78,79,80,81,82,83,84,85,86,87,88,89,90,91,92,93,94,95].

To facilitate researchers’ intuitive understanding of different characterization techniques, in addition to Table 1 which summarizes the fundamental information of each technique as presented above, the paper further compiles Table 2 to illustrate the various roles of these characterization techniques in studying different anode materials. The usage of these in situ characterization techniques in the development of anode materials for the three battery systems is summarized in Figure 2. At present, all eight types of in situ characterization techniques have been applied in the research of lithium-ion batteries. However, when studying sodium-ion batteries and potassium-ion batteries, some of these in situ characterization techniques are rarely involved, which demonstrates the mature application of in situ characterization technology in lithium-ion battery studies. Furthermore, the in situ XRD and in situ TEM techniques are applied more frequently in the three batteries compared to the other six in situ characterization methods, suggesting that in future studies, there is a significant potential to utilize different types of in situ characterization to further advance the study of different types of batteries.

Each in situ technique will be elaborated in following sections, focusing on their core advantages and illustrating their application in battery mechanism research and performance optimization with relevant research examples so as to provide support for the efficient application of in situ characterization techniques in the battery field.

### 2.1. In Situ XRD

In situ XRD enables real-time monitoring of lattice parameter and phase composition changes in the electrode or at the electrode–electrolyte interface during battery operation. Its critical applications in anode research include the following: (1) For intercalation-type anodes, it helps identify intermediate phase formation, clarify reaction pathways, and verify intercalation mechanisms and structural stability [85]. (2) For alloy-type anodes, it tracks alloying reactions and monitors volume expansion and structural degradation [86,96]. (3) For oxide-type anodes, it elucidates redox reaction mechanisms and assesses structural reversibility during cycling [87,97,98,99]. These insights provide essential theoretical support for structural optimization and performance enhancement of battery materials.

**Figure 3:**
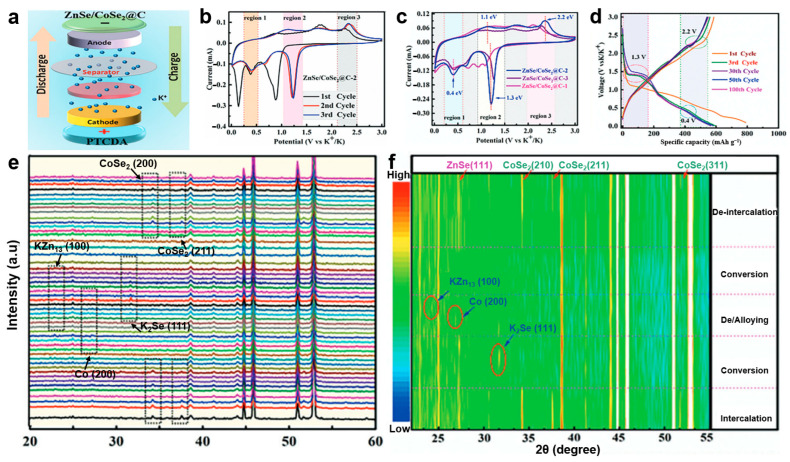
(**a**) Schematic model of the ZnSe/CoSe_2_@C-2||PTCDA full cell; (**b**) CV at different scan rates; (**c**) CV curves at a scan rate of 0.1 mV s^−1^; (**d**) charge/discharge curves for different cycles; (**e**,**f**) in situ XRD patterns of ZnSe/CoSe_2_@C-2. (Reproduced from [88] with permission from Wiley, 2024).

Iqbal et al. [88] synthesized a two-dimensional selenium-rich ZnSe/CoSe_2_@C heterostructure via hydrothermal and selenization methods as a high-performance anode for potassium-ion batteries (Figure 3a). Based on the CV and charge/discharge curve analysis (Figure 3b–d), the highly reversible potassium-ion insertion storage process in the heterostructure has been confirmed. However, to clarify the specific potassium storage mechanism, in-depth investigation using further characterization techniques such as in situ XRD is necessary. In situ XRD analysis (Figure 3e,f) provides further insight into the potassium storage mechanism. During discharge, the characteristic peaks of ZnSe and CoSe_2_ remain stable until 1.3 V, then gradually weaken and disappear, indicating the formation of K_x_ZnSe/CoSe_2_. A strong K_2_Se peak emerges at 0.8 V, reflecting the high crystallinity enabled by the heterointerface. At 0.01 V, new peaks corresponding to KZn_13_ and metallic Co confirm the alloying and reduction processes observed in CV. Upon charging, these intermediate phases disappear at 2.2 V, and the original ZnSe and CoSe_2_ peaks reappear and intensify at 3.0 V, demonstrating the excellent structural reversibility of the heterostructure anode.

In the aforementioned study, in situ XRD primarily served to monitor phase transitions in the electrode material during battery operation in real-time. However, its utility extends beyond alloy-type anodes and includes other anode categories, with capabilities surpassing mere phase tracking. A summary of these multifunctional applications is presented in Table 2, suggesting that in situ XRD characterization will play an important role in the study of charging/discharging processes of various batteries.

### 2.2. In Situ TEM

**Figure 4:**
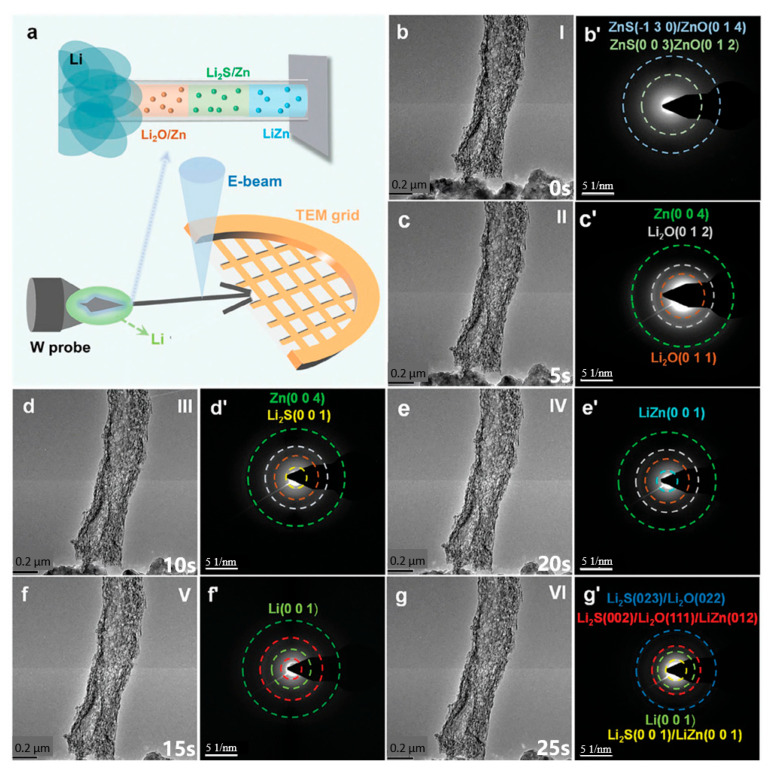
(**a**) Schematic illustration of the in situ TEM setup employed for the lithiation of ZOS-CF; (**b**–**g**) in situ TEM snapshots and (**b’**–**g’**) their corresponding SAED patterns of ZOS-CF captured at different times during the lithiation process. (Reproduced from [102] with permission from Wiley).

In situ TEM enables real-time observation of microstructural and interfacial evolution in electrode materials during electrochemical cycling, providing critical insights for designing high-performance anodes. A key challenge lies in accurately replicating the electrode’s operational state within the high-vacuum TEM environment [100]. Two primary approaches exist for environmental control. One is environmental TEM, which employs a differential pumping system to maintain a localized gas environment (Figure 4a). This configuration minimizes gas interference for high-resolution imaging yet is incompatible with liquid electrolytes. Alternatively, specialized in situ sample holders create a sealed electrochemical cell within the holder itself. This widely adopted method offers a low-cost use, compatibility with standard TEM instruments, and flexibility in experimental design.

For battery studies, two kinds of cell designs are commonly used: open-cell and closed-cell configurations. Open-cell setups provide higher spatial resolution and simpler fabrication but poorly simulate realistic battery conditions. In contrast, closed-cell systems enable compatibility with various electrolytes and facilitate nanomaterial observation in liquid environments, though they present greater technical challenges in implementation [89,101].

To gain deeper insights into the spontaneous reaction between ZnO/ZnS nanoparticles and lithium metal, Liu et al. [102] employed in situ TEM to characterize their lithiation process (Figure 4a). Throughout the entire lithiation process (Figure 4a–g), the carbon fibers exhibit no significant dimensional changes, indicating that the three-dimensional carbon-based scaffold can effectively mitigate volume expansion. Figure 4b,b’ presents the original TEM and the selected area electron diffraction (SAED) pattern of ZOS-CF prior to lithiation, respectively (Stage I), where two distinct diffraction rings are attributed to ZnO and ZnS. At the early lithiation stage (Stage II), new diffraction rings corresponding to the Zn phase and Li_2_O phase emerged in the SAED pattern (Figure 4c’), confirming the spontaneous reaction between lithium and zinc. With progressive lithiation (Stages III→V), the formation of Li_2_S and LiZn alloy evidences the conversion reaction and alloying reaction of ZnS (Figure 4d’,e’). After full lithiation (Stage VI), the diffraction rings in the SAED pattern (Figure 4g’) verify that ZOS-CF can spontaneously react with lithium metal to generate in situ-formed Li_2_O/Li_2_S phases and a LiZn alloy.

In situ TEM has proven effective in monitoring the microstructural evolution of anode materials in real-time, as demonstrated in elucidating the lithiation mechanism of molybdenum disulfide [103]. Moreover, this technique offers broader capabilities in studying other material systems, with specific applications summarized in Table 2.

### 2.3. In Situ EIS

In situ EIS serves as a powerful tool in anode material studies by monitoring dynamic interfacial changes during cycling. It captures critical parameters such as SEI film impedance and charge transfer resistance evolution. Through analyzing impedance variations at different cycling stages, this technique helps identify the primary causes of electrochemical performance degradation—whether originating from interfacial issues or deteriorated ion diffusion within the bulk material. Furthermore, in situ EIS enables quantitative comparison of interface impedance and charge transfer kinetics before and after material modification, providing direct evaluation of modification effectiveness and component synergy [90]. The acquired insights into SEI formation dynamics also guide the optimization of electrolyte composition, cycling protocols, and electrode fabrication processes, ultimately contributing to enhanced battery performance.

**Figure 5:**
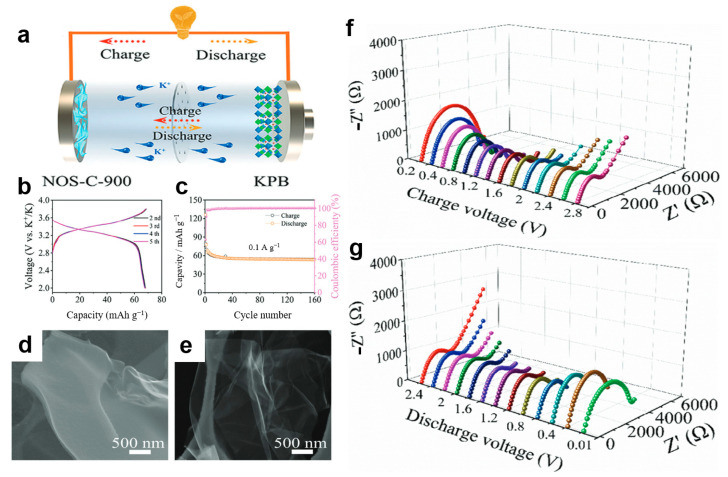
(**a**) Cell configuration to coin cell; (**b**,**c**) GCD curves and cycle performance of KPB cathodes at 0.1 A g^−1^; (**d**,**e**) SEM images of NOS-C-900 and NOS-C-1000; (**f**,**g**) in situ EIS plots of discharge and charge processes of the NOS-C-900 anode at 0.1 A g^−1^. (Reproduced from [104] with permission from Elsevier).

Xu et al. [104] employed in situ EIS to gain deeper insights into the potassium storage mechanism and electrochemical kinetics of the NOS-C-900 anode. The study begins by illustrating the battery configuration schematic (Figure 5a) and the electrode’s microstructure (Figure 5d,e). Figure 5f,g presents the in situ EIS response of the electrode during charge and discharge under a specified current density. The results indicate that as the discharge voltage drops into a lower range, the charge transfer resistance increases significantly, corresponding to the intercalation of a large number of potassium ions into the electrode. Conversely, during the charging process, this resistance shows a clear decreasing trend, indicating effective structural recovery of the electrode after depotassiation and confirming its highly reversible charge–discharge behavior. Further electrochemical performance (Figure 5b,c) further reveals the high reversibility and stability of the anode, providing circumstantial evidence for its excellent electrochemical kinetic performance and supporting the proposed surface–interlayer synergistic potassium storage mechanism.

In situ EIS fulfills distinct functions across different anode categories. For intercalation-type anodes, it primarily monitors SEI film stability and interfacial charge transfer resistance, thereby assisting in optimization of electrode fabrication processes [105]. For alloy-type anodes, it tracks impedance fluctuations induced by volume expansion and ion diffusion barriers, enabling verification of modification strategies [106]. For oxide-type anodes, it focuses on charge transfer resistance evolution during redox reactions and the impedance impact of newly formed interfaces, helping to differentiate the origins of performance degradation [107]. A summary of these multifunctional applications is presented in Table 2.

### 2.4. In Situ FTIR

In situ FTIR plays a vital role in anode material studies by enabling real-time tracking of functional group evolution during electrochemical processes. This capability provides critical insights into reaction mechanisms, including pathway identification and intermediate detection [108]. The technique effectively monitors characteristic functional group changes during solid electrolyte interphase (SEI) formation, determining its chemical composition and structural evolution across cycling stages to guide SEI optimization [109]. Furthermore, in situ FTIR assesses material stability through characteristic peak variations, detects structural degradation, and identifies side-reaction byproducts along with their triggering conditions, thereby contributing to enhanced battery performance and safety [110].

**Figure 6:**
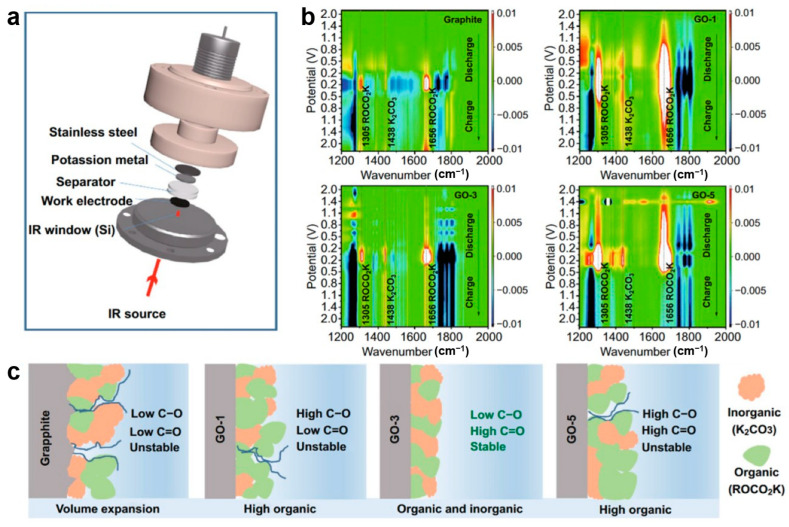
(**a**) Schematic representative of the in situ FTIR spectra electrochemistry cell; (**b**) FTIR spectra of graphite, GO-1, GO-3, GO-5; (**c**) schematic diagram of the SEI components (reproduced from [91] with permission from Springer).

Peng et al. [91] synthesized oxygen-doped graphite anodes for potassium-ion batteries and employed in situ FTIR to investigate how oxygen-containing functional groups enhance potassium storage. Using a specially designed in situ cell with a silicon crystal infrared window and MCT detector (Figure 6a), they characterize the electrode/electrolyte interface evolution of GO and graphite electrodes. The results reveal that oxygen functional groups catalyze SEI formation. On GO electrodes, SEI generation commences at ~2.0 V, evidenced by the decomposition of EC/DEC solvents appearing as new peaks at 1305, 1438, and 1661 cm^−1^ (Figure 6c), whereas graphite requires much lower potential (~0.8 V, Figure 6b). SEI thickness follows the order GO-1 > GO-5 > GO-3 > graphite, correlating with expansion rates at the 10th charge (12.0%, 6.7%, 5.5% vs. graphite). This trend aligns with improved initial coulombic efficiency. Mechanistically, C–O groups promote ROCO_2_K formation (most prevalent in GO-1), while C=O and COOH facilitate the generation of K_2_CO_3_. The latter contributes to a more stable and ion-conductive SEI. Combined with its relatively thin SEI, GO-3 demonstrates optimal rate capability and cycling stability. Notably, SEI component peaks attenuate but persist after charging, confirming dynamic stability in maintaining electrode integrity.

In situ FTIR provides distinct analytical capabilities for different anode material categories. For intercalation-type materials, it reveals ion intercalation mechanisms through functional group vibration shifts while simultaneously tracking the formation and dynamic evolution of the solid electrolyte interphase (SEI) [111]. For alloy-type materials, it monitors characteristic peak variations during alloying reactions to elucidate reaction pathways while capturing SEI damage and self-repair processes under volume fluctuation [36]. For conversion-type materials, it tracks oxygen-containing functional group transformations during redox processes to analyze reaction mechanisms while characterizing SEI composition and stability to support performance optimization [38]. A summary of these multifunctional applications is presented in Table 2.

### 2.5. In Situ Raman

In situ Raman spectroscopy plays a crucial role in anode material studies by enabling real-time monitoring of phase transitions, chemical bond evolution, and interfacial reactions under operational conditions [112]. This capability provides direct insight into lithium/sodium storage mechanisms, structural stability during cycling, and failure origins, thereby offering essential experimental evidence for optimizing the composition, structure, and performance of anode materials.

**Figure 7:**
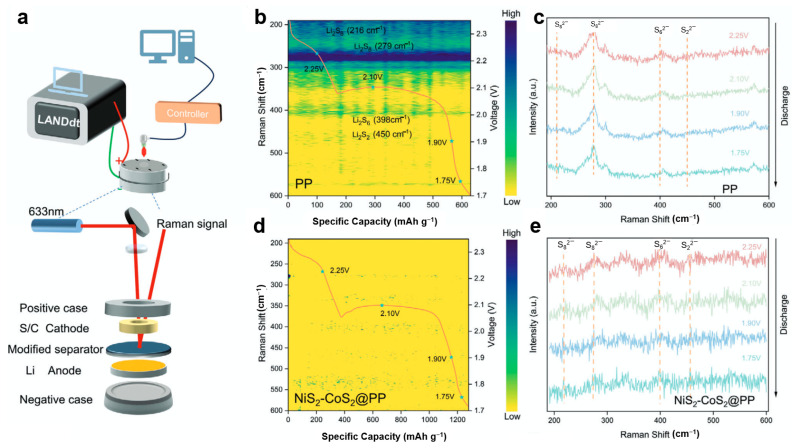
(**a**) Schematic diagram of the in situ Raman spectroscopy platform; (**b**,**c**) capacity-resolved Raman spectra and discharge profiles of the Li-S battery with PP separator; (**d**,**e**) capacity-resolved Raman spectra and discharge profiles of the Li-S battery with NiS_2_-CoS_2_@PP separator (reproduced from [113] with permission from Wiley).

To address the sluggish reaction kinetics in lithium–sulfur batteries, Ye et al. [113] developed a NiS_2_-CoS_2_ heterojunction material and employed in situ Raman spectroscopy to investigate its mechanism in suppressing polysulfide shuttling. Using a customized in situ platform integrating a Li-S cell with 633 nm laser Raman spectroscopy and an electrochemical workstation (Figure 7a), the researchers monitored polysulfide evolution under operational conditions. For cells with conventional PP separators (Figure 7b,c), discharge profiles and the corresponding Raman spectra reveal distinct signals at 216 and 279 cm^−1^ (long-chain Li_2_S_8_) during the first plateau. As the discharge progresses to the second plateau (~2.1 V), new peaks emerge at 398 and 450 cm^−1^ (Li_2_S_6_ and Li_2_S_2_), indicating significant polysulfide accumulation and shuttle effect. In contrast, cells with NiS_2_-CoS_2_@PP separators (Figure 7d,e) show barely detectable polysulfide signals throughout discharge, demonstrating the heterojunction’s effectiveness in capturing soluble polysulfides, accelerating redox kinetics, and preventing their migration across the separator.

In the development of intercalation-type, conversion-type, and alloy-type anode materials, in situ Raman spectroscopy serves the fundamental function of tracking microstructural evolution and chemical reactions in real-time during electrochemical cycling, thereby providing critical insights for performance optimization and failure analysis. Specifically, for intercalation-type anodes, it detects characteristic peak shifts during ion intercalation/deintercalation to assess reaction homogeneity and identify SEI components [114]. For conversion-type anodes, it monitors bond vibration changes to track metal ion redox states and structural phase transitions [115]. For alloy-type anodes, it follows characteristic peak evolution during alloying reactions to investigate structural degradation from volume expansion and cycle performance decay [116]. A summary of these multifunctional applications is shown in Table 2.

### 2.6. In Situ NMR

In situ NMR provides crucial insights into anode material behavior by directly tracking nuclear spin signal variations in guest ions under operating conditions. This technique enables real-time observation of ion diffusion pathways, site occupancy, and dynamic migration behavior. Simultaneously, it identifies material phase transformations, monitors electrode–electrolyte interfacial evolution, and quantifies ion populations in different states [46]. These capabilities deliver molecular-level evidence for elucidating storage mechanisms, correlating structural degradation with performance decay, and guiding rational material design.

**Figure 8:**
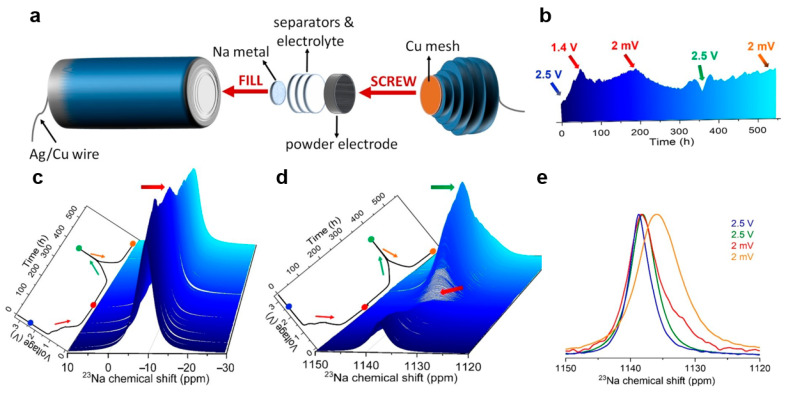
(**a**) Schematic representative of Na|NaPF_6_|HC cell for ^23^Na in situ NMR studies. (**b**) Projection of the enlarged 3D view of the ^23^Na in situ NMR spectra. (**c**) Enlarged 3D view of the ^23^Na in situ NMR spectra in the range of 10 to −30 ppm for the Na|NaPF_6_|HC cell. (**d**) Enlarged 3D view of the ^23^Na in situ NMR spectra in the range of 1150 to 1120 ppm. (**e**) Extracted and normalized ^23^Na in situ NMR spectra in the range of 1150 to 1120 ppm at the initial charge state of approximately 2.5 V, as well as at the highest voltage of 2.5 V and the lowest voltage of 2 mV (reproduced from [92] with permission from Wiley).

Šić et al. [92] developed an optimized cylindrical cell design for in situ ^23^Na solid-state NMR studies, particularly suitable for powder electrode materials (Figure 8a). Using hard carbon (HC) anodes and sodium metal reference electrodes, they conducted real-time NMR measurements during electrochemical cycling. Figure 8b,c illustrates the dynamic evolution of sodium ion behavior during cycling. The intensity of the electrolyte sodium ion signal varies progressively with the voltage, reflecting the ions’ reversible migration and indicating continuous interfacial reactions and SEI formation processes. Simultaneously, the corresponding signal changes in the hard carbon region further confirm the reversibility of the sodium storage mechanism within the electrode (Figure 8d,e). In this process, in situ NMR acts like a “real-time microscope,” allowing us to directly observe the dynamic migration of sodium ions during charge and discharge, as well as the ongoing chemical reactions at the electrode interface, thereby confirming the reversibility of the sodium storage mechanism within the battery.

For intercalation-type anodes, it tracks real-time ion (de)intercalation processes, site occupancy, and structural evolution, thereby revealing storage mechanisms and dynamic behavior [117]. For conversion-type anodes, it identifies reaction intermediates and products, clarifying redox pathways and interface transformation [118]. For alloy-type anodes, it monitors phase transitions, compositional changes, and local atomic environments during alloying reactions, helping us to understand reaction mechanisms and address challenges such as volume expansion. Collectively, these capabilities provide fundamental evidence for optimizing performance and elucidating reaction mechanisms across anode material systems. These multifunctional applications are summarized in Table 2.

### 2.7. In Situ XPS

In situ XPS provides critical insights into anode material behavior by enabling the real-time monitoring of surface and near-surface chemical states during electrochemical cycling. This technique tracks dynamic changes in elemental composition, chemical environment, and valence states, facilitating the investigation of ion intercalation mechanisms, solid electrolyte interphase (SEI) formation and evolution, and interfacial reaction dynamics. These capabilities help establish fundamental correlations between electrochemical performance and surface chemistry, thereby offering essential experimental support for optimizing anode material design and enhancing battery performance [44].

**Figure 9:**
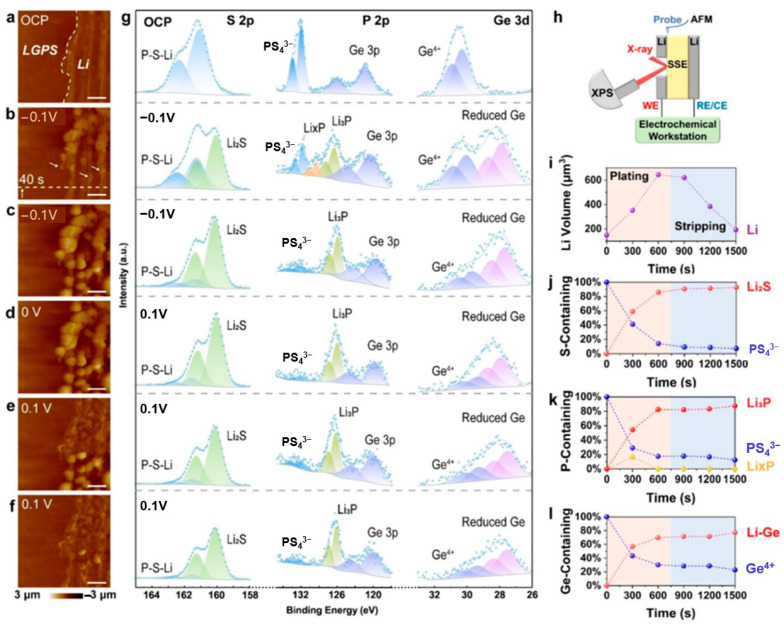
(**a**–**f**) AFM images: (**a**) Open Circuit Potential (OCP), (**b**,**c**) −0.1 V, (**d**) 0 V, (**e**,**f**) 0.1 V, with all scale bars being 4 μm and the scanning direction from bottom to top. (**g**) In situ XPS spectra of the Li-LGPS interface at different potentials, where the capture time of each AFM image and the collection time of each XPS spectrum are both 300 s. (**h**) Schematic diagram of the combination of in situ AFM and in situ XPS. (**i**–**l**) Changes in Li volume, S-containing species, P-containing species, and Ge-containing species during Li plating and stripping, respectively (reproduced from [119] with permission from Wiley).

Zhang et al. [119] employed coupled in situ AFM and XPS to investigate the morphological and chemical evolution at the Li-LGPS interface under low bias voltages. Figure 8 illustrates the key observations. At open circuit potential (OCP, Figure 9a), AFM identifies the Li-LGPS boundary while XPS (Figure 9g) primarily detects LGPS components. Upon applying a −0.1 V cathodic overpotential, particle nucleation occurs within 40 s (Figure 9b), evolving into dense Li deposition after 600 s (Figure 9c). Concurrent XPS analysis reveals the formation of a ternary-component SEI during deposition, as indicated by the increasing signals of Li_2_S and reduced Ge species, alongside the transient appearance of Li_x_P followed by sustained Li_3_P growth. During subsequent anodic polarization (+0.1 V), Li particles progressively dissolve (Figure 9d,e), leaving residual wrinkles after 600 s (Figure 9f). XPS spectra correlated with these morphological changes confirm the persistence of SEI components (Figure 9i–l). The experimental configuration (Figure 9h) featured a symmetric cell with precisely polished interfaces for simultaneous AFM morphology tracking and XPS chemical analysis. XPS quantification provides critical insights into the dynamic SEI composition. Throughout the deposition and stripping processes, the evolution of components such as Li_2_S, Li_3_P, and the Li-Ge alloy is clearly tracked, revealing the interface’s chemical transformation. In particular, XPS analysis demonstrates that the SEI maintains remarkable compositional stability during Li stripping despite the volume decrease, highlighting its interfacial robustness. This multimodal approach, as showcased in Figure 8, successfully correlates deposition kinetics with chemical evolution at the buried solid-state interface.

For intercalation-type anodes, it dynamically tracks binding energy shifts in host atoms and guest ions during deintercalation/intercalation, identifying insertion sites, elucidating chemical state evolution, and verifying reaction reversibility [120]. For conversion-type anodes, it enables real-time monitoring of transition metal redox behavior and lithium-containing product formation, helping validate conversion mechanisms and analyze irreversibility from incomplete reduction or side reactions [121]. For alloy-type anodes, it captures metal atom binding energy changes during alloying, detects irreversible byproducts, and reveals capacity fading mechanisms associated with irreversible phase transformations, thereby providing direct experimental evidence for optimizing material structure and performance [122]. A summary of these multifunctional applications is displayed in Table 2.

### 2.8. In Situ SANS

In situ SANS provides unique capabilities for investigating anode materials by exploiting differences in neutron scattering properties between electrolyte and electrode components. This technique quantifies ion concentrations within electrode pores through scattered neutron intensity measurements under varying potentials, enabling detailed analysis of ion adsorption/desorption processes [123]. Furthermore, it monitors ion behavior across different pore sizes, with enhanced sensitivity in smaller pores due to more pronounced scattering variations, offering valuable insights into pore-size-dependent storage mechanisms. However, since scattering signals depend on combined contributions from cations, anions, and solvent molecules, SANS faces inherent challenges in achieving fully quantitative speciation of pore ion populations [124].

**Figure 10:**
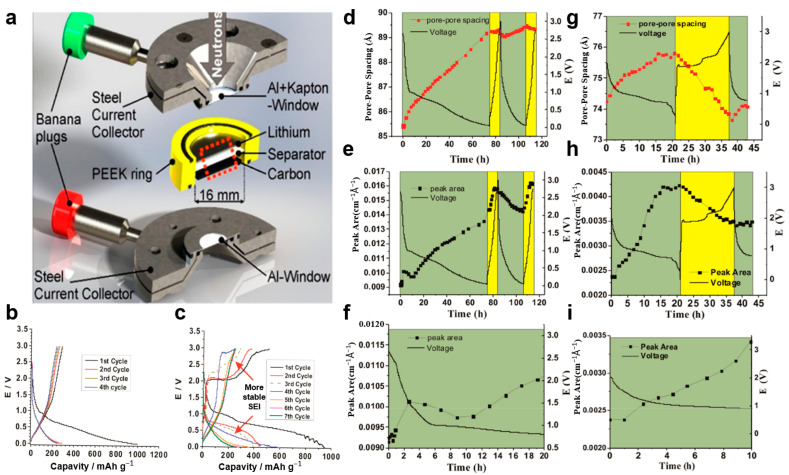
(**a**) Schematic view of the operando SANS cell (reproduced from [126] with permission from ACS Publications, 2019). (**b**,**c**) Charge−discharge profiles for ex situ half-cells under a current density of 25 μA cm^−2^. (**d**–**g**) In situ SANS data for cycling of half-cells containing ordered mesoporous hard carbon cathodes, ^7^Li anodes, and 0.5 M LiTFSI/MPPY·TFSI (**d**–**f**) or LiTFSI/EMIM·TFSI (**g**–**i**) electrolyte. Darker background for discharge regions, lighter background for charged regions; (reproduced from [125] with permission from ACS Publications).

Bridges et al. [125] employed in situ SANS to investigate the stabilization mechanism of high-capacity hard carbon during initial cycling in ionic liquid electrolytes (Figure 10a). A comparative analysis of hard carbon electrodes in EMIM·TFSI and MPPY·TFSI electrolytes, via SANS measurements, reveals distinct differences in their interfacial evolution. As shown in Figure 10d–f, the MPPY·TFSI system exhibits a marked increase in peak area during initial discharge, corresponding to electric double layer formation and Li^+^ desolvation/intercalation processes within specific voltage windows. In contrast, the EMIM·TFSI system shows a similar but weaker response (Figure 10g–i). This divergence is attributed to cation-specific behavior: both EMIM^+^ and MPPY^+^ participate in co-intercalation or reduction reactions. Obviously, the MPPY·TFSI system demonstrates more pronounced SEI formation in the first cycle, with its coulombic efficiency rapidly stabilizing at a high level by the fifth cycle (Figure 10b,c). These SANS observations align with previously reported solvated Li^+^ adsorption behavior in carbonate electrolytes, confirming that variations in scattering length density directly reflect changes in Li^+^ concentration within the carbon pores. Thus, this study provides nanoscale structural evidence for understanding the interfacial chemical evolution of hard carbon across different ionic liquid environments.

In situ SANS has exceptional sensitivity to light elements and the unique capability of being able to probe buried interfaces, making it particularly valuable for elucidating complex electrochemical mechanisms. By correlating structural parameters with electrochemical performance, in situ SANS enables the fundamental understanding of degradation pathways and facilitates the rational design of advanced electrode architectures with enhanced stability and kinetics. The technique’s non-destructive nature and statistical sampling capabilities further distinguish it from other characterization methods in battery research. A summary of these multifunctional applications is presented in Table 2.

### 2.9. Application Limitations of In Situ Characterization Techniques

Although individual in situ characterization techniques have played important roles in various battery studies, their application still has some limitations. Table 3 summarizes the current contributions and limitations of various in situ characterization methods. More practical issues still need to be taken into consideration when selecting and employing different in situ characterization techniques for specific applications.

In situ characterization techniques exhibit distinct trade-offs in sample preparation complexity, cost, and applicability for alkali metal ion battery anode studies. In situ XRD demands precise control of electrode thickness and low-absorption window materials with moderate preparation costs and low testing expenses, making it ideal for batch dynamic monitoring of crystalline phase transitions. In situ TEM requires advanced micro–nano cell packaging to reconcile vacuum conditions with liquid electrolytes, leading to high preparation and testing costs with relatively low sample success rates and expensive equipment maintenance. In situ EIS features low preparation complexity and cost, as it only requires stable electrode–electrolyte interfaces and leak-free cell packaging, enabling long-term impedance tracking at minimal expense. In situ Raman spectroscopy relies on flat electrode surfaces and light-transmitting windows, with moderate preparation costs and low testing fees, suitable for surface component dynamics. In situ FTIR faces challenges from infrared light penetration limits and brittle window materials, resulting in slightly higher preparation costs than Raman and moderate testing costs, with strict humidity control required during analysis. In situ NMR necessitates non-magnetic cell packaging and high-purity electrolytes to avoid signal distortion under strong magnetic fields, leading to high preparation costs and extremely expensive testing due to the large-scale nature of NMR equipment. In situ XPS requires ultra-high vacuum-compatible cells and an inert atmosphere transfer to prevent electrode oxidation, with high preparation and testing costs stemming from vacuum system maintenance and long pumping times. In situ SANS depends on optimized scattering contrast between electrodes and electrolytes, as well as low-background packaging, with moderate preparation costs but prohibitive testing expenses due to limited access to synchrotron radiation or neutron sources.

To address the current issues, a significant amount of research and technological breakthroughs are still required, such as reducing the manufacturing cost of the testing devices, innovating and developing new materials for key devices, designing and developing in situ testing systems based on new working principles, etc. It is believed that through continuous efforts, costs and technical limitations in the application of in situ characterization technology will be continuously overcome. In the future, in situ characterization techniques will play a more widespread role in the laboratory research and industrial application of Li-, Na-, and K-ion batteries.

### 2.10. Combined Use of In Situ Characterization Techniques

**Figure 11:**
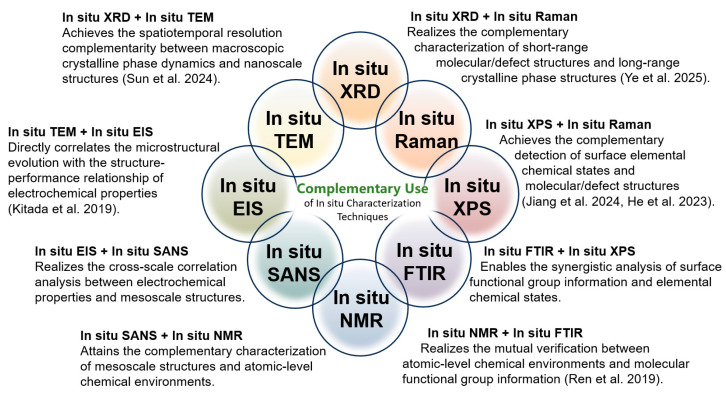
Combination utilization of different in situ characterization techniques [46,86,113,127,128,129].

A single in situ characterization technique obtains limited detection information during its use. If we want to fully and comprehensively reveal the various details changes during the battery charging/discharging process from multiple perspectives, the combined use of two in situ characterization techniques is a very effective strategy. Several complementary application schemes for in situ characterization techniques are proposed in Figure 11, some of which have been validated by successful cases in relevant studies, suggesting their important role in revealing the new principles and phenomena of battery reactions.

In situ XRD + in situ TEM: The core advantage of the combination of in situ XRD and in situ TEM is that it achieves the complementarity of spatiotemporal resolution between macroscopic crystalline phase dynamics and nanoscale structures. With this combined approach, it is possible to simultaneously reflect the dynamic change information of macroscopic crystalline phases and the structural characteristics at the nanoscale, enabling researchers to observe the dynamic processes of material structures across different scales. It is reported that when Cu_2_S@NSC is used as an anode for sodium-ion batteries, in situ XRD tracks the evolution of the crystal structure during cycling, confirming the reversible conversion reaction between Cu_2_S, Cu, and Na_2_S. While in situ TEM directly presents the morphological changes in nanosheets, the sodium insertion process, and the volume expansion buffering effect of the carbon layer, verifying that structural integrity is maintained. This compensates for the lack of macro phase laws or the micro intuitiveness in single characterization, providing comprehensive and rigorous experimental support for explaining the mechanism of the material’s excellent sodium storage performance [110].

In situ XRD + in situ Raman: The advantage of the combination of in situ XRD and in situ Raman lies in its complementary characterization of short-range molecular/defect structures and long-range crystalline phase structures, thus completing the correlation analysis of material structures at different scales. In the study of potassium storage in graphite foam, in situ XRD tracks the long-range crystal structure evolution of K-GICs, confirming reversible stage transitions and identifying the intermediate phase KC_16_. In situ Raman characterizes short-range charge transfer and local structural changes, revealing the transformation in the middle stage. This process makes up for the limitations of single characterization and offers rigorous support for explaining the potassium storage mechanism [87].

In situ TEM + in situ EIS: When Fe_3_N@NCNT is used as an anode for Li-ion batteries, in situ TEM directly observes that Fe_3_N nanoparticles are effectively encapsulated by NCNTs, with the composite structure remaining stable after long-term cycling, while reference samples show particle detachment or pulverization. At the same time, in situ EIS quantifies its electron/ion transport efficiency and stability, confirming excellent electrochemical performance [46]. Their core complementarity lies in the following aspects: TEM directly presents the microscopic phenomenon of “structural stability” and EIS quantifies the electrochemical result of “superior performance”, directly correlating “NCNT encapsulation induced structural stability” with “low impedance and high diffusion efficiency induced excellent cycling/rate performance”. This compensates for the lack of intuitiveness or quantifiability in single characterization, making the demonstration of the material’s advantages more rigorous.

Recently, the combined application of two or more in situ characterization techniques has been increasingly adopted in various battery studies. With the complementary use of various in situ characterization techniques, more and more new discoveries will be revealed during the electrochemical charging and discharging process of batteries. With the continuous technological updates and the reduction in manufacturing costs, this combined in situ characterization strategy will be adopted more widely in the future.

## 3. Summary and Perspectives

In situ characterization techniques are indispensable for understanding the complex electrochemical processes within rechargeable batteries, providing real-time insights into structural and chemical evolution during operation [130]. This review systematically examines their application in anode materials for a range of alkali metal ion batteries (AMIBs), including Li-, Na-, and K-ion battery systems [131,132,133]. These in situ characterization techniques can facilitate the elucidation of the related electrochemical mechanisms. Individual in situ techniques play distinct roles. In situ XRD tracks crystalline phase transitions, TEM visualizes nanoscale structural evolution, EIS quantifies interfacial impedance, and Raman/FTIR probes chemical bond changes, while NMR, XPS, and SANS complement these methods with insights into ion transport, surface chemistry, and nanoscale morphology. However, each method has limitations. Synergistic use of techniques overcomes single-method drawbacks, enabling comprehensive understanding of anode behavior. These advances facilitate anode optimization, yet future efforts should address extreme condition adaptability and intelligent data analysis to further advance battery technology. For future development, the following perspectives are proposed.

1. Advancing characterization capabilities for complex operational scenarios remains a critical challenge. Under extreme battery conditions—such as high voltage (>4.5 V) and wide temperature ranges (−40 °C to 80 °C)—current in situ setups often suffer from limited stability and signal interference. Future development should focus on designing electrochemical cells resistant to high-voltage corrosion, detection systems with minimized thermal drift, and enhanced data correction algorithms. These improvements will mitigate environmental interference and expand the applicability of in situ techniques under realistic operating conditions.
2. Advancing intelligent characterization represents a crucial direction for evolution. Current in situ methodologies predominantly rely on manual data processing, leading to inefficiencies and potential oversight of critical information. Future development should integrate artificial intelligence to establish comprehensive data systems capable of real-time characterization, automated data acquisition, intelligent analysis, and mechanistic prediction.
3. Developing more robust and versatile in situ cells that are compatible with diverse characterization techniques while accurately mimicking the internal environment of practical batteries is a key direction for obtaining scientifically relevant and industrially applicable insights [96,134].
4. There is a critical need to bridge the gap between model materials and practical cells by applying these advanced in situ diagnostics directly to commercially relevant electrodes under realistic cycling conditions, including high mass loading, limited electrolyte, and wide voltage windows.
5. Besides its application in the field of anode materials, in situ characterization technology can also play a significant role in other research areas of batteries. The application of in situ characterization techniques in artificial and biomimetic protective coatings will provide real-time insights into the dynamic evolution of coating microstructures, interfacial interactions, and degradation processes under service conditions. This will deepen the understanding of structure–function relationships and guide the rational design of high-performance, long-life protective coatings [135,136].

## Figures and Tables

**Table 1:** Resolution, characterization information, and physical parameters of in situ techniques.

In Situ Characterization	Spatial Resolution	Temporal Resolution	Information				Physical Parameters
			Morphology	Structure	Chemistry	Kinetics	
In situ XRD	≈μm	60 ms	—	√	—	—	Unit cell parameters, crystal phase content, etc.
In situ TEM	0.1 nm	10 ns	√	√	√	—	Particle size, interplanar spacing, etc.
In situ EIS	nm–μm	1–5 s	—	—	√	√	Electrochemical parameters including impedance and capacitance
In situ Raman	10 nm–1 μm	1–5 ms	—	√	√	—	Vibration mode parameters
In situ FTIR	1–10 mm	1–5 s	—	√	√	—	Absorption peak intensity/position, etc.
In situ NMR	0.1–1 μm	1–10 ms	—	√	√	√	Chemical shift, relaxation time, etc.
In situ XPS	0.05–10 nm	3–10 s	—	—	√	—	Binding energy, atomic concentration, etc.
In situ SANS	≈nm	30–60 s	√	√	—	—	Particle size, dispersibility, etc.

**Table 2:** The specific roles of several in situ characterization techniques in the development of anode materials for Li-, Na-, and K-ion batteries.

In Situ Characterization Technique	Anode Materials	Function	Ref.
In situ XRD	CMPTO	Monitor the phase transition during the electrode reaction process	[25]
	SnSb@C	Reveal the crystal structure changes in the material during the K-ion insertion process	[26]
	Fe_3_N@C	Uncover the growth and preparation process of materials	[27]
	(FeMnNiCuZn)_3_O_4_	Analyze the stress and strain caused by the volume expansion of the material	[28]
In situ TEM	EGaIn@C	Observe the changes in microstructure, mechanical behavior, and phase transformation process in real-time	[29]
	Mn_2_P@NPC	Explore the interaction of nanoparticles in nanomaterials	[30]
	Ni_3_S_2_	Monitor and explore the changes in mechanical behavior and microstructure	[31]
	Sb@CNFs	The real-time observation of the alloying/dealloying reaction process confirms that the carbon shell ensures the structural integrity of the material	[32]
In situ EIS	Co_9_S_8_@CoWO_4_	Prove that the material can stably form a stable SEI film in the ester electrolyte	[33]
	TMPSe	Monitor the formation, growth and evolution of the SEI film in real-time	[34]
In situ FITR	MoN_x_	Testing of surface and interface reactions and adsorption and reactions on material surfaces	[35]
	CPs	Research on the relationship between functional groups and performance	[36]
	ALTO@C	The monitoring of material synthesis and preparation processes involves inferring reaction mechanisms based on changes in chemical bonds	[37]
	Si (with PAA)	Disclose the conformational and chemical structural changes that occur during the manufacturing process of the electrode materials	[38]
In situ RAMAN	ZnMn-NC/GF	Reflect the shuttle behavior of poly-halogen intermediates (polyiodides/polybromides) on different separators	[39]
	BO-CP	Analyze the internal chemical bonds, crystal structure, and other structural information of the material	[40]
	Hard Carbon (HC)	Reveal the insertion and storage mechanism of sodium ions in hard carbon	[41]
	N-GCNs	Reveal the “adsorption mechanism” for Na storage and the “adsorption-intercalation mechanism” for Li storage	[42]
In situ XPS	Sb_2_O_3_/rGO	Monitor the elemental composition and valence state changes in the anode material	[43]
	NiFe_2_O_4_/CoFe_2_O_4_	Reveal the significant advantages of the Na storage mechanism compared to Li storage mechanism.	[44]
In situ NMR	rGO	Real-time monitoring of the intercalation and deintercalation processes of lithium ions in electrode materials	[45]
	SiO	Reveal the formation mechanism of the lithium silicide phase on silicon monoxide	[46]
In situ SANS	Hard Carbon	Monitor ion diffusion	[47]
		Monitor the changes in the interface structure	
	Metallic Lithium	Interface reaction monitoring	[48]

**Table 3:** Summary of contributions and limitations of in situ characterization techniques.

In Situ Characterization	Contributions	Restrictions	Battery Setup/Configuration
In situ XRD	Structure information of crystal phase	1. It is hard to distinguish signals from electrode and interphase	Coin cell, pouch cell, “coffee bag” cell, 18,650 cell, AMPIX cell, Swagelok cell, RATIX cell
		2. Amorphous or organic phase information is unattainable	
In situ TEM	Morphology and crystalline interplanar spacing information	1. High-energy electron beam dose may destroy the sample	Open-cell configuration, sealed-cell configuration, liquid cell configuration
		2. It is hard to observe the swelling or opaque sample	
In situ EIS	Interface impedance evolution; ion transport kinetics; electrode/electrolyte reaction resistance change	1. Hard to distinguish individual impedance components precisely	Coin cell, pouch cell, three-electrode cell, Swagelok cell
		2. Low sensitivity to fast dynamic reaction processes	
In situ Raman	Structure and bond information of both crystal and amorphous phase	1. The Raman shift may be influenced by the fluorescence effect	Three-electrode cell, coin cell, pouch cell
		2. The high-energy laser may burnout the sample	
In situ FTIR	Chemical bond information of organic species	1. The results may be affected by liquid electrolytes and air	Internal reflection configuration, external reflection configuration
		2. It is hard to analyze inorganic species	
In situ NMR	Structural information of crystal and amorphous	1. Time-consuming analysis procedure leads to low temporal resolution	Thin-walled NMR-compatible cell, non-magnetic sealed cell, custom glass cell, three-electrode electrolytic cell
		2. The skin effect/skin depth may interfere with the test	
		3. Magnetic components may interfere with the signal	
In situ XPS	Chemical bond information of surficial species	1. Conventional XPS requires UHV condition	Thin-window pouch cell, three-electrode vacuum cell
		2. It is not sensitive to low-conductivity species	
In situ SANS	Nanoscale structure and interface microstructural evolution; Li^+^ transport-related morphological changes	1. Low spatial resolution, poor local heterogeneity capture	Thin-walled pouch cell, quartz capillary cell, sealed transmission cell
		2. Long exposure time, limited fast dynamic response	
		3. Sensitive to sample thickness/crystallinity	

## Data Availability

No new data were created or analyzed in this study. Data sharing is not applicable to this article.
